# From implementation to discontinuation: multi-year experience with the multiple sclerosis performance test as a digital monitoring tool

**DOI:** 10.3389/fdgth.2025.1672732

**Published:** 2025-10-03

**Authors:** Dirk Schriefer, Anja Dillenseger, Yassin Atta, Hernan Inojosa, Tjalf Ziemssen

**Affiliations:** Center of Clinical Neuroscience, Department of Neurology, Medical Faculty and University Hospital Carl Gustav Carus, TUD Dresden University of Technology, Dresden, Germany

**Keywords:** Multiple Sclerosis Performance Test (MSPT), implementation science, user experience, digital monitoring, patient-reported experience measures (PREM), acceptability, usability, patient-centered care

## Abstract

**Introduction:**

Digital tools such as the self-administered Multiple Sclerosis Performance Test (MSPT) support structured monitoring of multiple sclerosis (MS) through standardized assessments of motor, visual, and cognitive functions. Despite clinical validity and adoption, real-world data on long-term user experiences and the consequences of discontinuing MSPT-based monitoring in routine care are lacking.

**Objective:**

This study aimed to assess multi-year user experiences with the MSPT among patients and neurologists, investigate patient perceptions following its discontinuation from clinical care, and evaluate preferences for future MSPT-like digital tools.

**Methods:**

This observational, repeated cross-sectional study involved three questionnaire-based surveys. In 2020, separate surveys of patients and neurologists (combined *n* = 210) evaluated sustained MSPT use in routine care. Following the cessation of funding and subsequent discontinuation of MSPT from clinical workflows in 2023, a patient survey was conducted in 2024 (*n* = 144) to evaluate the impact of this withdrawal and preferences for future digital monitoring tools. Quantitative analyses included frequency distributions, Net Promoter Score (NPS) categorization, correlational analyses, and descriptive data visualization.

**Results:**

Patients reported high satisfaction with MSPT usability, utility for disease monitoring, administration frequency, time efficiency, physical and cognitive demands, and suitability for unsupervised tablet-based use. Most viewed discontinuation from their clinical care negatively and favored reintroducing similar tools, either in clinic (85.5%) or at home (78.6%). Those who dissented cited time savings and sufficient physician feedback.

**Discussion:**

Prolonged MSPT use is associated with strong patient and clinician acceptance. Findings support the continued integration of digital monitoring tools into MS care and emphasize the importance of patient perspectives in their design.

## 1 Introduction

Multiple sclerosis (MS) is a chronic, immune-mediated demyelinating disease of the central nervous system with a wide range of clinical manifestations (1). Over time, MS leads to accumulating disability, diminished quality of life, and substantial socioeconomic impact (2). Common symptoms include impaired ambulation, upper extremity dysfunction, cognitive deficits, visual disturbances, fatigue, and mental health issues, among others. Regular disease monitoring is essential for effective disease management, allowing for timely assessment of disease progression, symptom fluctuations, and treatment efficacy (3–5).

Advances in digital health technologies have enhanced the efficiency of monitoring MS by enabling structured, longitudinal data collection in a resource-optimized manner (6–10). Digital tools for symptom assessment and disease management range from passive monitoring approaches, such as wearable sensors and digital symptom-tracking tools that facilitate continuous data collection in real-world settings, to active monitoring approaches utilizing standardized, performance-based assessments administered either remotely or in traditional clinical care settings (11–13). A key example of the latter is the Multiple Sclerosis Performance Test (MSPT), an iPad® (Apple Inc., Cupertino, CA, USA)-based, self-administered disability assessment battery that digitizes established analog neuroperformance tests to evaluate key domains of motor, visual and cognitive function (8, 14).

The MSPT represents a major advance in MS assessment, addressing key limitations of traditional clinical disability measures (15). Based on the Multiple Sclerosis Functional Composite (MSFC), it improves on the clinician-driven Expanded Disability Status Scale (EDSS), which is an ordinal scale that overvalues ambulation while neglecting cognition, vision, and upper limb function, and has limited rater consistency (16–18). The four component MSFC (MSFC-4) addresses these gaps by incorporating performance-based, quantitative measures - the Timed 25-Foot Walk (T25FW), the Nine-Hole Peg Test (9HPT), the Paced Auditory Serial Addition Test (PASAT) and the Sloan Low-Contrast Letter Acuity Test (LCLA) - to provide a more objective assessment of MS-related multidomain disability (17, 19, 20). However, the MSFC is limited by its reliance on in-person administration and manual data collection, resulting in variability in test conditions, high time and personnel requirements, and challenges in scaling its use, making it difficult to implement on a regular basis (15). The self-administered MSPT overcomes these limitations with digital technology standardizing and automating data collection, enabling real-time data processing, and facilitating the integration with electronic health records and research databases (21). The MSPT also includes digital versions of validated patient-reported outcome measures that capture perceived disability and multiple dimensions of Quality of Life in Neurological Disorders (Neuro-QoL) through electronic questionnaires (22, 23).

The MSPT was a key component of the MS Partners Advancing Technology and Health Solutions (MS PATHS) initiative, a multicenter learning health system funded by Biogen International GmbH and launched in 2015 (24). Expanded to a global network of ten healthcare facilities, MS PATHS collected standardized, multidimensional data for patient care and research purposes, with MSPT assessments systematically incorporated into routine clinical evaluation. However, despite demonstrating value in MS monitoring, this large-scale MSPT data collection was prematurely terminated in June 2023 following the cessation of MS PATHS funding.

Prior to its discontinuation, studies had established the MSPT's validity and reliability (25–27), as well as its feasibility (demonstrated by quantitative time metrics and completion rates) (21, 28), and usability (supported by qualitative patient satisfaction data) (14, 21). However, user-centered evaluations were limited to the early stages of MSPT implementation and did not examine long-term real-world experiences of patients and clinicians utilizing the MSPT over the course of years in routine care. This represents a critical knowledge gap, as sustained engagement with digital health tools often declines over time due to unmet usability needs, workflow misalignment, or diminished perceived value - a well-documented trend in implementation science (29–31). Longitudinal user experience evaluations that encompass domains such as usability, acceptability, user satisfaction, and perceived impact possess the capacity to identify persistent barriers to effective use and to guide strategies to improve the sustained uptake of digital tools such as the MSPT (29–32).

Furthermore, patient perspectives on the abrupt discontinuation of the MSPT from clinical care have not been evaluated so far, leaving unanswered questions about its patient perceived impact on disease management.

To address existing knowledge gaps, this survey study investigated patient experiences with the MSPT during two key phases: (i) its active use in routine clinical practice and (ii) the period following its discontinuation. The aim was to generate real-world insights into prolonged use of the MSPT and to inform future strategies for digital MS monitoring. Specifically, the study pursued three objectives:

- To assess patient (and physician) experiences and perceptions of MSPT implementation, including perceived burden, convenience, ease of use, need for support, perceived value for disease monitoring, feasibility of independent use, and preferences regarding frequency and timing.
- To examine how patient characteristics influence these experiences, with the aim of identifying potential barriers and facilitators to implementation.
- To understand patient perspectives on the MSPT's discontinuation, including perceived impacts on MS management and expectations for future digital monitoring tools.

## 2 Materials and methods

This study evaluated patient and clinician experiences with the MSPT as a whole, with a focus on the neuroperformance modules (addressing cognition, vision, upper/lower extremity function) and the Neuro-QoL (addressing physical, mental, and social health).

### 2.1 Study design and population

A repeated cross-sectional survey study was conducted at the MS Center Dresden (Germany) during two distinct phases: (i) during active implementation of the MSPT following nearly three years of integration into routine clinical workflows (Substudy 1, 2020), and (ii) over 1.5 years after its 2023 discontinuation from clinical use (Substudy 2, 2024).

In Substudy 1 (2020), patients enrolled in the MS PATHS program were invited to complete a paper-based patient-reported experience measure (PREM) questionnaire during routine visits (October 2019–April 2020), immediately following in-clinic MSPT administration. Eligible participants had a confirmed diagnosis of MS or clinically isolated syndrome (CIS), were ≥18 years old, physically able to perform the MSPT, and had provided written informed consent as part of the MS PATHS 888MS001 study (Ethics approval: EK58022017). In April 2020, treating neurologists were additionally surveyed regarding their experiences with MSPT use. Substudy 1 was initiated as a local quality improvement initiative to identify barriers to sustained MSPT integration.

In Substudy 2 (2024), eligible patients - aged ≥18 years, diagnosed with MS or CIS, and with prior MSPT experience - were invited to complete an anonymous web-based survey. Recruitment was conducted via email newsletters and podcast outreach (https://zkn.uniklinikum-dresden.de/en/pn). The eligibility of subjects was verified through embedded screening questions.

### 2.2 Patient and physician questionnaires

#### 2.2.1 Patient survey outcomes

In 2020 (Substudy 1), patients completed an 11-item paper-based questionnaire that assessed various aspects of the patient experience, including task burden, ease of completion, quality of support, perceived utility for disease monitoring, feasibility of independent use, and preferences for frequency and timing. Most items used 11-point ordinal scales (0–10), while one item used a three-point scale (Supplementary Table S1).

In 2024 (Substudy 2), an anonymous web-based questionnaire was administered after discontinuation. The included seven core items on the impact of MSPT withdrawal, interest in MSPT-like tools, and preferences for future digital monitoring. All items were categorical (Supplementary Table S2).

#### 2.2.2 Physician survey outcomes

In Substudy 1, physicians completed a 14-item questionnaire reflecting patient survey domains, adapted to the clinician perspective. Responses primarily used 11-point scales, with a single item using a three-level scale (Supplementary Table S3).

### 2.3 MSPT implementation and outcomes

From 2017 to June 2023, the MSPT was deployed as part of the MS PATHS initiative at the Dresden MS Center. Patients typically completed the iPad®-based assessment quarterly during routine visits, with the Neuro-QoL modules administered biannually to reduce the burden on patients. Standardized audiovisual tutorials guided self-administration.

To optimize local implementation, two enhancements were introduced:

- Patients received real-time result summaries and could request support from study staff during testing. A study nurse ensured task completion and facilitated discussions. If scores showed clinically meaningful declines (e.g., ≥20%), neurologists were notified.
- Following evidence of clinical utility, MSPT was extended beyond the MS PATHS cohort to all patients in the MS Center Dresden, with locally stored data informing clinical care.

#### 2.3.1 Neuroperformance testing and outcomes

The MSPT included four self-administered functional tests:

- Walking Speed Test (WST): Assesses lower limb function via timed 25-ft walks, derived from the T25FW (25, 33). Outcome: time in seconds.
- Processing Speed Test (PST): Measures cognitive speed through symbol-digit matching under time pressure, adapted from the SDMT (27, 34). Outcome: number of correctly matched pairs
- Manual Dexterity Test (MDT): Evaluates upper limb function by placement/removal of nine pegs from an integrated board, based on the 9HPT (25, 35). Outcome: completion time per hand in seconds.
- Contrast Sensitivity Test (CST): Assesses visual function using high (100%) and low (2.5%) contrast letter recognition, based on the LCLA (20, 25). Outcome: number of correctly identified letters

Raw scores were converted to z-scores based on normative data (25, 36).

#### 2.3.2 Neuro-QoL testing and outcomes

The Neuro-QoL component assessed patient-perceived physical (fatigue, mobility, hand function), mental (anxiety, depression, stigma, cognition), and social health (role participation/satisfaction) via computer adaptive testing. Domain scores were reported as standardized T-scores (22, 37, 38).

#### 2.3.3 Other outcomes

Substudy 1 obtained MSPT outcomes, module completion times, and Patient-determined Disease Steps (PDDS) scores from the MS PATHS research portal (22). Additional clinical variables, including EDSS, were extracted from medical records. Substudy 2 relied solely on self-reported data obtained through the anonymous online survey, which prevented linkage to clinical or retrospective MSPT data. Both substudies collected five core patient variables: age, sex, disease duration, MSPT experience, and a disability measure.

### 2.4 Statistics

Quantitative variables were summarized using the arithmetic mean with standard deviation (SD), median with interquartile range (IQR: 25th–75th percentile), or the 5th–95th percentile range, as appropriate. Categorical variables were reported as absolute and relative frequencies.

Questionnaire responses on the 11-point scale were visualized with 100% stacked bar charts to show the full response distributions. Further, measures of central tendency and dispersion were reported, treating the data as approximately interval. Item scores were further categorized into detractors (0–6, reflecting less favorable responses), neutrals (7–8), and promoters (9–10, reflecting most favorable responses), following Net Promoter Score (NPS) methodology. The NPS was calculated as the percentage of promoters minus the percentage of detractors (range: −100 to +100), offering a conservative group-level summary measure (39, 40). Originally created for single-item customer satisfaction scales, it was applied here to all experience questionnaire items.

A Principal Component Analysis (PCA) was performed on the 14 patient questionnaire items to reduce dimensionality and identify key domains of patient experience. The analysis used the Kaiser criterion, Cattell's Scree Test, and varimax rotation, yielding a four-component solution explaining 61% of the total variance. Suitability for PCA was confirmed via Bartlett's test of sphericity (*p* < 0.05) and the Kaiser-Meyer-Olkin measure (KMO > 0.70). The extracted components (domains) were labeled: (1) Task Strain, (2) Ease & Independence, (3) Outcome Utility & Timing, and (4) Support & Comfort (see Table 1 for component loadings).

**Table 1:** Summary statistics and principal component analysis results of the patient questionnaire (*n* = 200).

Questionnaire items (item number)	Central tendency and dispersion		Percentage distribution					Principal components (loadings and labels)			
	Median (5–95th PCT); IQR	Mean ± SD	0–6 (%)	7–8 (%)	9–10 (%)	NPS	N	PC1: Task strain	PC2: Task ease & independence	PC3: Outcome utility & timing	PC4: Support & comfort
Low physical/cognitive strain during MSPT (q8)	10 (3–10); 8–10	8.75 (2.15)	9.6%	19.8%	70.6%	60.9%	197	**0.920**	0.149	−0.050	0.127
Low physical/cognitive strain during Neuro-QoL (q9)	10 (2–10); 8–10	8.63 (2.51)	14.7%	11.7%	73.6%	58.9%	197	**0.821**	0.335	0.031	−0.059
Low physical/cognitive strain during NPTs (q10)	10 (3–10); 8–10	8.73 (2.20)	11.7%	18.3%	70.1%	58.4%	197	**0.900**	0.066	0.024	0.148
Ease of performing the MSPT (as a whole) (q1)	10 (5–10); 9–10	9.04 (1.69)	6.0%	18.5%	75.5%	69.5%	200	0.234	**0.819**	−0.026	0.031
Ease of completing the Neuro-QoL (q2)	10 (5–10); 9–10	9.01 (1.75)	7.5%	16.1%	76.4%	68.8%	199	0.096	**0.783**	−0.003	−0.059
Ease of performing the NPTs (q3)	10 (7–10); 9–10	9.29 (1.43)	4.5%	13.0%	82.5%	78.0%	200	0.043	**0.635**	0.108	0.262
Suitability of MSPT for independent conduct (q11)	10 (8–10); 9–10	9.58 (1.06)	1.0%	9.1%	89.9%	88.9%	198	0.107	**0.648**	0.057	0.051
Personal use of MSPT results for self-monitoring (q4)	8 (0–10); 5–10	6.89 (3.67)	35.5%	15.0%	49.5%	14.0%	200	−0.019	0.052	**0.541**	0.296
Usefulness of results for clinical MS monitoring (q5)	10 (5–10); 9–10	8.91 (2.08)	11.0%	13.5%	75.5%	64.5%	200	−0.078	0.127	**0.727**	0.007
Conviction of results use in clinical MS monitoring (q6)	10 (5–10); 8–10	8.85 (2.00)	12.0%	15.0%	73.0%	61.0%	200	−0.088	0.156	**0.535**	0.175
Appropriateness of MSPT time requirement (q7)	9 (3–10) 7.5–10	8.32 (2.22)	20.0%	20.5%	59.5%	39.5%	200	0.262	−0.044	**0.675**	−0.177
Appropriateness of quarterly MSPT conduct (0, 5, 10)^a^ (q14)	5 (0–5); 0–5	3.81 (2.30)	0^a^: 25.3%	5^a^: 73.2%	10^a^: 1.5%	n.a.	198	0.015	−0.135	**0.725**	0.008
Comfort of MSPT implementation (q12)	10 (6–10); 8–10	8.95 (1.55)	7.1%	22.7%	70.2%	63.1%	198	0.101	0.060	0.195	**0.735**
Good support & explanations by study staff (q13)	10 (9–10); 10–10	9.79 (0.68)	1.0%	3.5%	95.5%	94.4%	198	0.065	0.076	−0.029	**0.668**

Patient experience questionnaire results from Substudy 1. Items are grouped by conceptual domains, with principal-component loadings from the four-component solution shown in the final four columns. Primary loadings (all >0.500) are highlighted in bold and there are no significant cross-loadings (all <0.350). The components (domains) are labeled as follows: (1) Task Strain, (2) Task Ease & Independence, (3) Outcome Utility & Timing, and (4) Support & Comfort.

IQR, interquartile range; MS, multiple sclerosis; MSPT, Multiple Sclerosis Performance Test; Neuro-QoL, Quality of Life in Neurological Disorders; NPS, Net Promoter Score; NPTs, neuroperformance tests; PC, principal component; Pct, percentile; SD, standard deviation.

Bold values indicate the primary component loadings (>0.500) of each questionnaire item on the respective principal component.

^a^ Measured on a 3-point ordinal scale (0 = Less frequent, 5 = Sufficient, 10 = More frequent) instead of the standard 11-point scale used for all other questionnaire items.

Bivariate associations between patient experience items and patient characteristics were examined in both substudies. In Substudy 1, Spearman's rank correlation coefficients were used for ordinal data. In Substudy 2, phi and point-biserial coefficients were applied for binary outcomes. Effect sizes were interpreted as small (*r* = 0.10), medium (*r* = 0.30), or large (*r* = 0.50) (41). Associations were further explored using regression analyses. Substudy 1 used linear regression models with the four PCA-derived experience domain scores as dependent variables. Substudy 2 applied logistic regression models to key binary survey items. The same five harmonized patient characteristics - age, sex, disease duration, MSPT experience, and disability - which were available in both samples, were included as categorical predictors in the regression models.

## 3 Results

### 3.1 Population characteristics

The Substudy 1 cohort consisted of 200 eligible people with MS (pwMS) (97.6% of 205 screened) and 10 physicians, with a mean patient age of 43.8 ± 11.7 years. PwMS were predominantly female (79.5%) and had relapsing-remitting MS (92.5%). The median EDSS score was 2.0 (IQR 1.5–3.75), indicating mild to moderate disability (Supplementary Table S4). Participants had completed a median of five MSPT assessments (IQR 3–6), representing up to 2.5 years of longitudinal MSPT testing experience.

Overall, the completion rate for each neuroperformance test was at least 98%. The full neuroperformance battery (CST, PST, WST, MDT) took an average of 16.50 ± 7.47 min to complete, while the Neuro-QoL took 6.58 ± 3.58 min. The module-specific completion times for the CST, PST, WST, MDT and Neuro-QoL, along with the test results, are detailed in Supplementary Table S5.

The Substudy 2 cohort included 144 eligible pwMS (92.9% of 155 screened). While the sex distribution was comparable to Substudy 1 (*p* = 0.935), participants were significantly older (*p* < 0.05), a difference attributable to the 5-year interval between both substudies (age-adjusted *p* = 0.766). This patient cohort had up to six years of MSPT experience. Detailed population characteristics are provided in Supplementary Table S4.

### 3.2 Patient experience survey (substudy 1)

**Figure 1:**
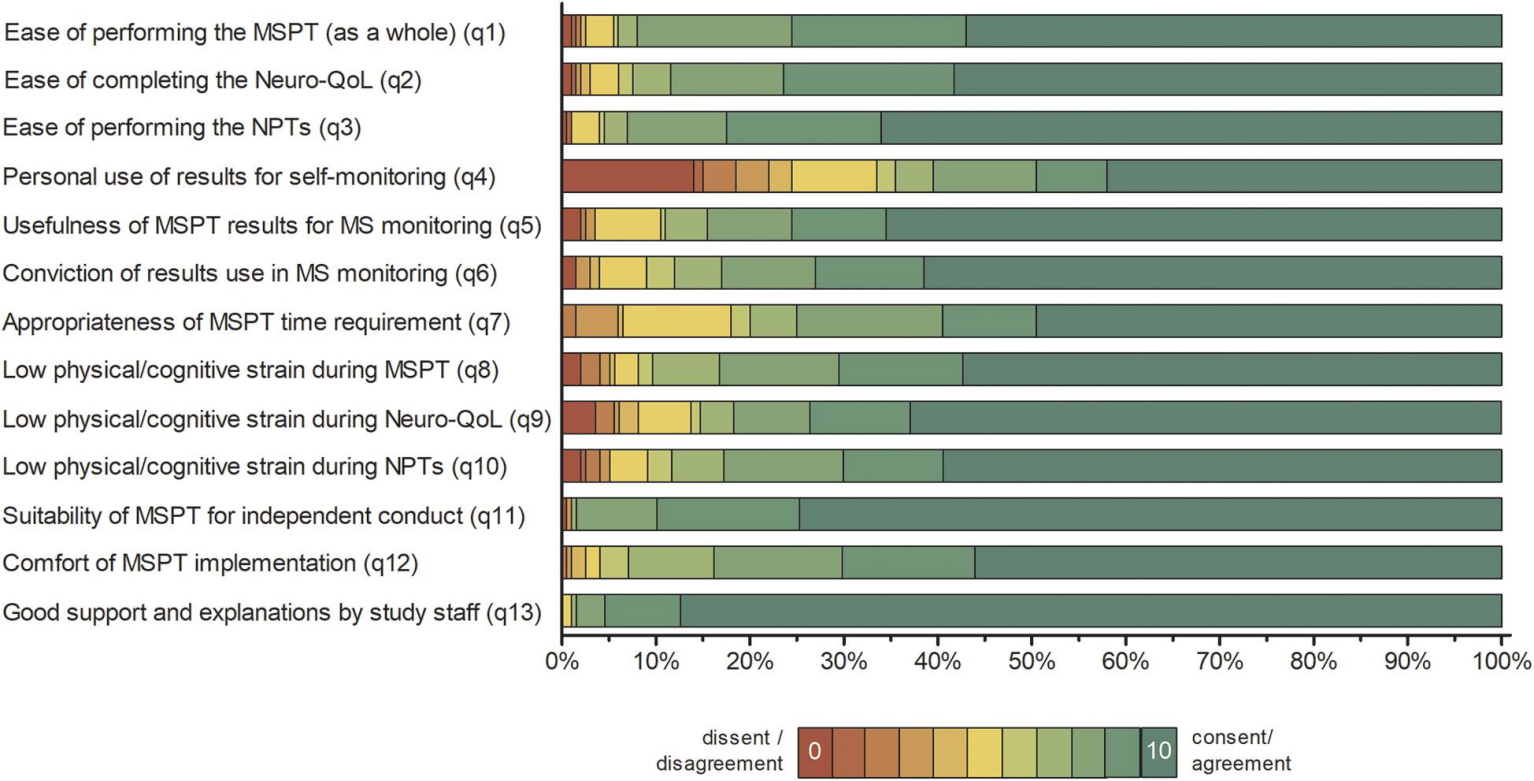
Distribution of patient responses to the MSPT experience questionnaire (*n* = 200). Percentage distribution of patient responses presented as 100% stacked bar charts, showing all individual response categories for items 1 to 13 without aggregation. Item responses are colour coded from red (lowest agreement, value = 0) to green (highest agreement, value = 10). Full patient questionnaire item wording is available in Supplementary Table S1. Summary statistics and principal component analysis results are provided in Table 1. NPT, neuroperformance test; Neuro-QoL, quality of life in neurological disorders; MSPT, Multiple Sclerosis Performance Test.

Overall, patient responses revealed predominantly favorable experiences across all domains, with ratings concentrated at the upper end of the 0–10 scale and median values typically in the 8–10 range. Figure 1 illustrates the percentage distribution of responses for each questionnaire item.

#### 3.2.1 Task strain

The majority of patients reported low levels of (physical/cognitive) strain during the MSPT, with 70.1% (neuroperformance tests) to 73.6% (Neuro-QoL) rating their experience in the promoter range of 9–10 (Figure 1 and Table 1; items 8–10, means >8.5).

#### 3.2.2 Ease and independence

PwMS rated the MSPT highly for perceived ease of use, with 75.5% (MSPT overall) to 82.5% (neuroperformance tests) assigning scores of 9–10, reflecting positive usability feedback on aspects such as the comprehensibility of the instructions, readability, and clarity of the questions on the iPad® interface (Figure 1 and Table 1; items 1–3, means >9). Furthermore, the ability to complete the MSPT independently and without support was endorsed to a considerable extent, with approximately 90% of the participants providing promoter ratings that contributed to the second highest NPS of all items (item 11, means >9.5).

#### 3.2.3 Outcome utility and timing

Patients expressed strong confidence in the overall usefulness of MSPT results for disease monitoring and their effectively use by physicians for disease management, with three out of four pwMS providing promoter ratings (Figure 1 and Table 1; items 5 & 6, means >8.5). With regard to patient's self-monitoring practices, the use of MSPT results for personal disease self-management exhibited the highest range and dispersion among all items. Nearly half of pwMS were promoters, while one-third were detractors (rating 0–6), resulting in the lowest NPS (item 4, mean 6.89 ± 3.67). The next lowest NPS appeared for the time burden item, with nearly 60% of patients rating the time investment as highly acceptable (scores of 9–10), but about 20% of detractors finding the duration of the MSPT to be too long (Table 1; item 7). Regarding preferred testing frequency, around three-quarters of pwMS favored quarterly MSPT assessments, while the remainder indicated a preference for less frequent testing (Table 1 and Figure 1; item 14).

#### 3.2.4 Support and comfort

Staff support received the highest NPS among all survey items, with 95.5% of respondents classified as promoters - reflecting an exceptionally high level of satisfaction with clinical staff (Table 1; Item 13, mean >9.5). The overall comfort experienced during MSPT administration was also predominantly positive, with around 70% of pwMS providing promoter ratings and a further 23% reporting more neutral scores in the 7–8 range (Figure 1 and Table 1; item 12, mean of approximately 9).

#### 3.2.5 Determinants of patient experience

**Figure 2:**
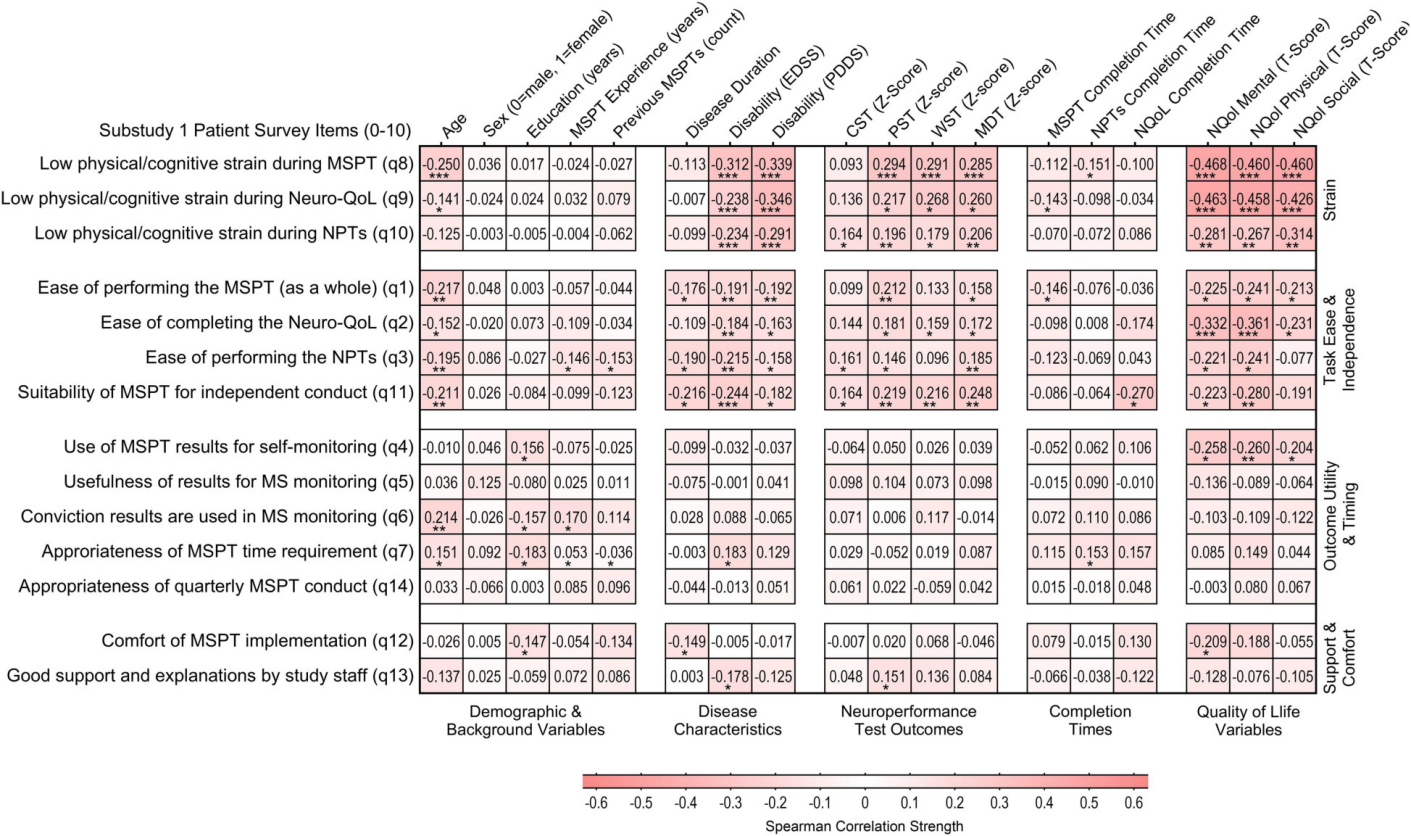
Association between patient characteristics and patients’ experience and perceptions of the MSPT (*n* = 200). Heatmap illustrating correlation patterns (Spearman's rho) between MSPT patient experience items and sociodemographic characteristics, disease-related factors, and MSPT outcomes, organized by PCA-derived domains. Higher z-scores for CST, PST, WST, and MDT indicate better neuroperformance. Higher scores for Neuro-QoL Mental, Physical, and Social composite domains, derived as averages of the associated Neuro-QoL domain T-scores, indicate worse self-reported health or impairment. Mental domains include depression, anxiety, stigma, and cognitive function. Physical domains encompass upper and lower extremity function, sleep, and fatigue. Social domains include participation in social roles and satisfaction with social roles (Supplementary Table S5; Figure S1 for details). Statistically significant associations are marked with asterisks: *p < 0.05, **p < 0.01, ***p < 0.001. NPT, neuroperformance test; NQoL, quality of life in neurological disorders (Neuro-QoL); MSPT, Multiple Sclerosis Performance Test.

Correlations between PREM questionnaire items and patient characteristics, including demographic factors, disease-related variables, and MSPT outcomes, were generally small to moderate (Figure 2). The most notable associations were observed for items in the task strain (PC1) and task ease & independence (PC2) domains. Advanced age and higher disease burden (indicated by greater disability scores, poorer neuroperformance test results, and lower Neuro-QoL scores) showed modest yet largely significant associations with increased task strain, reduced task ease, and lower perceived suitability of the MSPT for independent conduct. In contrast, items from the outcome utility & timing (PC3) and support & comfort (PC4) domains demonstrated consistently weaker, mostly nonsignificant associations in both item-wise correlation and linear regression analyses using domain summary scores (Figure 2 and Supplementary Figure S1 and Table S6). Notably, neither patients' cumulative MSPT experience nor the total completion time for the MSPT modules significantly influenced PREM responses.

### 3.3 Physician experience survey (substudy 1)

**Figure 3:**
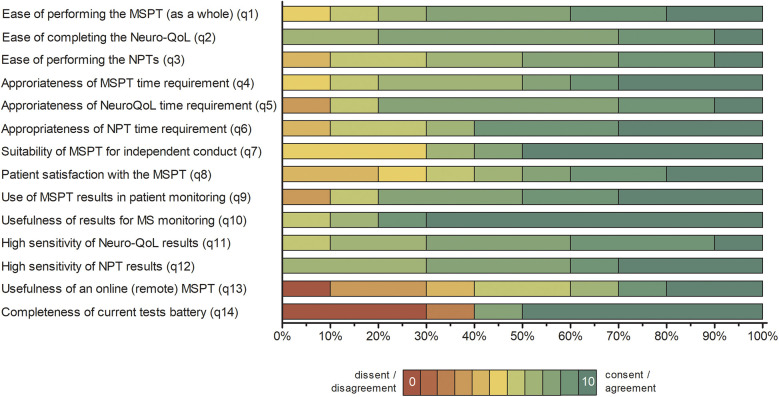
Distribution of physician responses to the MSPT experience questionnaire (*n* = 10). Percentage distribution of physician responses presented as 100% stacked bar charts, displaying all individual response categories for items 1–14 without aggregation. Item responses are color-coded from red (lowest agreement; 0) to green (highest agreement; 10). Full questionnaire item wording and summary statistics are provided in Supplementary Table S2. NPT, neuroperformance test; Neuro-QoL, quality of life in neurological disorders; MSPT, Multiple Sclerosis Performance Test.

Physician responses indicated predominantly favorable experiences, with ratings clustered at the upper end of the scale and median ratings between 6 and 10, though on average marginally lower than patient ratings (Figure 3 and Supplementary Table S2). Physicians strongly endorsed the ease of use of the MSPT (items 1–3), the suitability of independent administration of the MSPT (item 7), and the appropriateness of time requirements (items 4–6 and 14). They also endorsed the clinical value of the MSPT, including its utility for MS monitoring (items 9–10) and result sensitivity (items 11–12), with MSPT results' usefulness for disease management receiving the highest NPS (item 10). However, some reservations were noted regarding remote administration of the MSPT (item 13) and the completeness of the test battery (item 15; Figure 3 and Supplementary Table S2).

### 3.4 Patient survey on MSPT discontinuation (substudy 2)

Following the discontinuation of regular MSPT testing as part of clinical routine, a significant number of pwMS (89/144; 61.8%) reported negative impact related to omission. The main negative impact was the perceived loss of important disease monitoring data (97.8%), followed by reduced contact with study staff (29.2%) and the need to rely on alternative monitoring tools, such as apps or diaries (Table 2). Overall, the vast majority of pwMS (123/144, 85.4%) supported the resumption of regular MSPT-like digital monitoring. Of these, two thirds (67.5%) expressed a preference for maintaining the original MSPT format. Suggested improvements included more tests variety (27.6%), incorporating gamification elements (22%) and further adaptations to address MS-specific limitations (14.6%).

**Table 2:** Patient perspectives on MSPT discontinuation and future monitoring preferences (*n* = 144).

Item	*n*	%
**Negative impact of MSPT discontinuation (q1)**	(*n* = 144)	(100%)
▪ Yes	89	61.8%
▪ No	55	38.2%
Reasons for the negative impact of MSPT discontinuation (if q1 = yes; q2a)	(*n* = 89)	(100%)
▪ Absence of key feedback for validating self-monitoring of the disease	87	97.8%
▪ Lack of contact with study staff	26	29.2%
▪ Need to rely on alternative monitoring options (apps, diaries)	6	6.7%
▪ Others	1	1.1%
Reasons for no impact (or positive impact) of MSPT discontinuation (if q1 = no; q2b)	(*n* = 55)	(100%)
▪ Never used MSPT data for personal self-assessment	13	23.6%
▪ Doctor's feedback is sufficient (or more important)	38	69.1%
▪ Shorter visits are more valuable	10	18.2%
▪ MSPT was too demanding	5	9.1%
▪ Perceived pressure to succeed and stress	10	18.2%
▪ Other reasons	9	16.4%
**Interest in resumption of regular MSPT-like monitoring^a^ (q3)**	(*n* = 144)	(100%)
▪ Yes	123	85.4%
▪ No	21	14.6%
Suggestions/requests for resumption of MSPT-like monitoring^a^ (if q3 = yes; q4a)	(*n* = 123)	(100%)
▪ The format should remain the same	83	67.5%
▪ More gamified testing desired	27	22.0%
▪ Lower time commitment preferred	10	8.1%
▪ More variety in the tests can be beneficial	34	27.6%
▪ Adaptions for limitations needed (e.g., alternative or language-based assessments)	18	14.6%
Reasons for lack of interest in resumption of MSPT-like monitoring^a^ (if q3 = no; q4b)	(*n* = 21)	(100%)
▪ Time commitment is too demanding	11	52.4%
▪ Tests & questionnaires are too stressful	4	19.0%
▪ No regular MS confrontation desired	13	61.9%
▪ Other reasons	2	9.5%
Considering self-monitoring^a^ under certain conditions (if q3 = no; q4c)	(*n* = 21)	(100%)
▪ Yes	5	23.8%
▪ No	16	76.2%
**Role of support to perform self-monitoring^a^ during routine visits (q5)**	(*n* = 144)	(100%)
▪ Proper explanations make self-monitoring feasible for all patients.	85	59.0%
▪ Support level similar to the MSPT-period is ideal	29	20.1%
▪ Support upon request would suffice	79	54.9%
▪ Others	0	0%
**Willingness to conduct regular digital self-monitoring^a^ at home (remote) (q6)**	(*n* = 144)	(100%)
▪ Yes	113	78.5%
▪ No	31	21.5%
Acceptable frequency of remote (home-based) digital self-monitoring^a^ (if q6 = yes; q7a)	(*n* = 113)	(100%)
▪ Weekly	25	22.1%
▪ Every two weeks	20	17.7%
▪ Monthly	49	43.4%
▪ Less than monthly	18	15.9%
▪ Others	2	1.8%
Barriers to home-based digital self-monitoring^a^ (if q6 = no; q7b)	(*n* = 31)	(100%)
▪ Time commitment is too high	10	32.3%
▪ Difficult to integrate into daily routine	10	32.3%
▪ Tests & questionnaires are too stressful.	6	19.4%
▪ No regular MS confrontation desired	18	58.1%
▪ Concern about data security and privacy	3	9.7%
▪ Own control over access to data and test results wanted	5	16.1%
▪ Internet connectivity issues	5	16.1%
▪ Others	8	25.8%

Percentage distribution of responses to the post-MSPT-discontinuation survey. Multiple selections were permitted for non-dichotomous items, whereas yes/no items required a single response. Full questionnaire wording is provided in Supplementary Table S3.

^a^ Monitoring refers to the regular, self-administered completion of computerized MSPT-like neuroperformance tests (e.g., walking speed, manual dexterity, processing speed, visual acuity) and digital questionnaires (e.g., quality of life) used by patients to track symptoms and disease progression in domains such as mobility, dexterity, cognition, and vision.

Regarding the potential for home-based self-assessment, over three-quarters of pwMS (113/144, 78.5%) expressed willingness to participate in such a remote approach. The majority of participants (84.2%) preferred remote testing more frequently than once per month, largely exceeding the schedule used during the MSPT's active in-clinic implementation phase.

Among those who either perceived no negative impact of discontinuation (55/144, 38.2%), were not in favor of resuming MSPT-like digital monitoring (21/144, 14.6%), or declined home-based remote testing (31/144, 21.5%), the most commonly cited reasons were: trust in the (non-MSPT) feedback from their physicians, which they felt was sufficient for disease management; not interested in using MSPT results for patient self-monitoring; time savings by not having to undergo the MSPT; avoidance of regular MS confrontation; and stress associated with testing (Table 2).

### 3.5 Determinants of patient experience (substudy 2)

**Figure 4:**
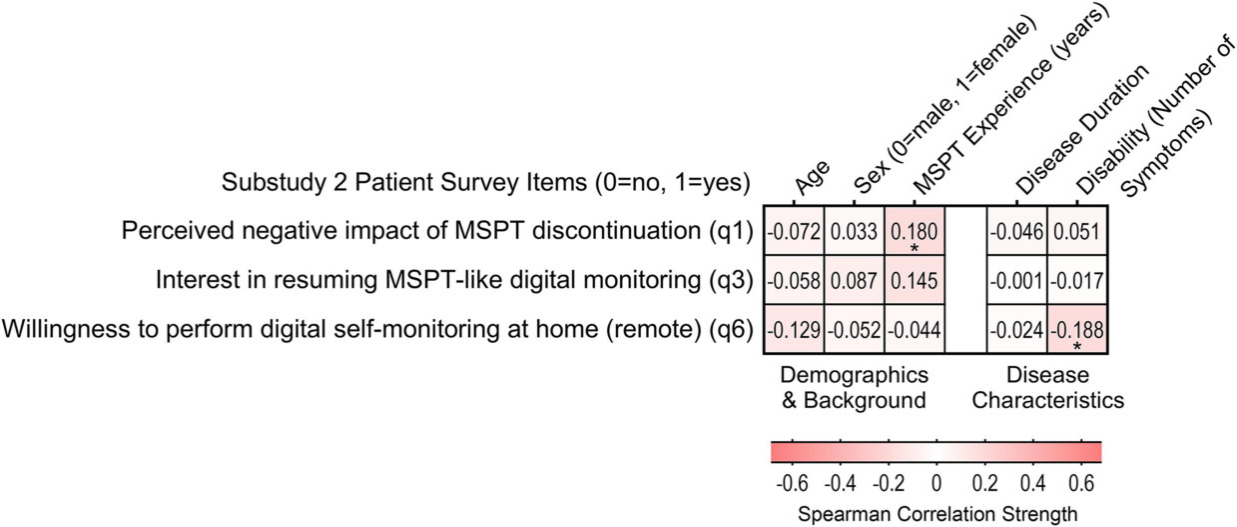
Association between patient characteristics and patients’ experience and perceptions of MSPT discontinuation and future monitoring preferences (*n* = 144). Heatmap showing bivariate correlations (phi and point-biserial correlation coefficients) between binary outcomes related to MSPT discontinuation (negative impact of MSPT discontinuation, interest in continued MSPT-like in-clinic monitoring, and interest in future remote monitoring) and sociodemographic as well as disease-related factors (Supplementary Table S4). Statistically significant associations are marked with asterisks: * *p* < 0.05.

The associations between patient characteristics and MSPT discontinuation-related outcomes - whether participants perceived a negative impact, wished to continue MSPT-like digital monitoring in the clinic, or were open to remote testing - were all negligible to minimal. The only trend observed, albeit modest, was a slightly lower willingness to undergo remotely tests among participants with a higher disability burden (Figure 4 and Supplementary Table S7).

## 4 Discussion

This study explored long-term user experiences with the MSPT during routine MS care and following its discontinuation. It captured insights from patients who had engaged with the MSPT for up to six years, as well as perspectives from their treating physicians. Overall, both groups reported predominantly positive experiences and satisfaction with the MSPT.

The key findings revealed that the MSPT imposed acceptable physical and cognitive demands, with task burden and completion times rated as easily manageable overall. The tool was perceived as easy to administer and suitable for independent use without direct supervision. Further, patients strongly endorsed the clinical utility of MSPT results for physician-led monitoring, although views were more reserved regarding the self-use of the results for their own disease management.

The present findings address critical gaps in longitudinal, user-centered evaluation and demonstrate sustained usability, utility, acceptability, and patient satisfaction with the MSPT beyond its earlier clinical implementation (14, 21, 26, 29) - factors essential for the long-term viability of digital health tools. The positive experiences and perceived benefits may promote sustained patient participation and help mitigate the decline in engagement often seen with the prolonged use of digital assessment tools (29–32, 42–44). Collectively, these results support the value of ongoing technological monitoring in MS care and align with other empirical findings on the long-term acceptability of contemporary digital monitoring tools in MS, as shown in recent research studies (42, 45, 46).

Since its inception, the MSPT/MS PATHS programme has emphasized the patient voice through advisory boards and other qualitative feedback channels (24). The present study not only corroborates the positive patient perceptions captured in two early patient satisfaction surveys (14, 21), but also extends existing MSPT user experience research in significant ways. First, it provides evidence on multi-year patient experiences with the fully-integrated MSPT in real-world clinical practice, moving beyond earlier implementation phases (14, 21). Second, this work also incorporates clinician experiences and perspectives, offering a more comprehensive understanding (29, 47). Third, including a European patient cohort may improve the generalisability of the findings, given that previous patient experience survey studies have been conducted in US-based research settings. Fourth, this study strengthens methodological rigor by utilizing a larger sample size and more comprehensive questionnaire items with expanded response scales compared to prior user experience evaluations (14, 21). Finally, our study is the first to examine the patient-perceived effects of discontinuing the MSPT after its long-term implementation in clinical practice, following the cessation of MS PATH funding.

The importance of evaluating long-term patient and physician perspectives on MSPT use is further underscored by the early termination of two related studies (NCT04599023, NCT04326637), which aimed to assess the MSPT's practical feasibility using qualitative satisfaction measures and quantitative engagement metrics. By addressing these evidence gaps - particularly from the real-world viewpoint of patients and clinicians - this study provides timely and relevant insights.

Findings from Substudy 2 suggest that discontinuing the MSPT after years of clinical integration was perceived negatively by most patients. Some, however, viewed the discontinuation as beneficial, citing reduced time burden and decreased emotional strain associated with confronting their disease. Despite these differences, a clear majority expressed strong interest in continuing digital monitoring and showed openness to both in-clinic and remote MSPT-like tools. These results reflect the considerable value patients place on digital monitoring in the context of their ongoing care.

Correlational analyses demonstrated consistent patient experiences across key demographic and clinical subgroups. Age, sex, disease duration, and prior MSPT experience were not significant barriers to positive evaluations of utility, timing, support, or the perceived consequences of MSPT discontinuation. However, older age and greater disease burden - reflected in EDSS, PDDS, Neuro-QoL, and MSPT performance metrics - were associated with an increased perception of task strain and a reduced ease of test completion. These findings underscore the importance of providing optional, personalized support, especially for older patients and individuals with greater functional limitations, to promote equitable access to and sustained engagement with digital health technologies (48, 49).

In addition to the subjective user experiences captured through patient and physician questionnaires, the high completion rates of MSPT modules and efficient administration times observed in this study (Supplementary Table S5) provide objective evidence supporting the sustained real-world feasibility of the MSPT, corroborating earlier findings. The mean completion time for all four neuroperformance test modules (the sum of the WST, MDT, PST, and MDT) was 16.50 ± 7.47 min, which is consistent with or slightly faster than the times reported in earlier studies of clinical implementation phases. Those times ranged from 17.05 to 18.01 min for the fully integrated MSPT (21, 28). These slightly reduced completion times likely reflect increased user familiarity with the MSPT in our cohort, beyond the effects of initial learning - an aspect that has been previously linked to faster testing (21, 28). Of note, MDT completion times were not faster than in earlier studies, likely because most of the testing time is devoted to the task itself rather than skippable instructions or setup procedures. Furthermore, the MSPT neuroperformance test scores in our cohort were slightly higher, and the study population was younger than in previous reports. These factors may have contributed to slightly shorter completion times (21, 28). Nevertheless, given the age and sex distributions (Supplementary Table S4), our cohort remains representative of a typical MS population, albeit from a specialized MS center.

This study was initially designed as a single-phase investigation focused on the active implementation of the MSPT (Substudy 1). Following the termination of Biogen-sponsored MS PATHS funding in 2023, the scope was expanded to include Substudy 2 in order to address emerging questions related to the impact of MSPT discontinuation. This extension adhered to established standards for observational research and transparent reporting (50).

Despite these methodological strengths, the study's findings should be interpreted in light of several limitations. First, the patient and physician experience questionnaires were self-developed and not formally validated. Due to the exploratory and context-specific nature of the study, as well as the absence of suitable, validated instruments, a pragmatic tool was designed to capture real-world user feedback. The questionnaire was developed with input from researchers experienced in digital health implementation and was adapted from other in-house MS patient experience instruments that cover functional assessments such as gait, jump, virtual reality, and speech tasks (51–53). Although the absence of formal psychometric validation remains a methodological limitation, PCA of the patient questionnaire items revealed clearly interpretable domains with minimal cross-loadings. This supports the structural validity of the instrument beyond face validity. Second, the highly positive patient ratings may reflect a ceiling effect due to limited discrimination of the 11-point Likert scale. Future work may use more psychometrically validated, sensitive instruments to capture finer gradations in user experience and rule out scale-related bias. Third, due to the anonymous design of Substudy 2, individual-level data linkage across the two survey phases was not possible. As a result, the extent of overlap between the two patient cohorts - and thus the number of unique participants - remains unknown. However, a comparative analysis of demographic characteristics revealed no significant differences between the two groups. Fourth, although the patient sample sizes were robust (*n* = 200 and *n* = 144 for Substudies 1 and 2, respectively), only ten neurologists completed the physician survey. Nevertheless, this number reflected the complete population of treating neurologists at the study site during Substudy 1 and therefore ensured full institutional representation. Fifth, the generalizability of the findings is limited by the single-center design. Our study setting is a specialized MS clinic characterized by a high rate of disease-modifying therapy use (90%) and relatively low disability levels (median EDSS 2), which differs from typical community-based MS cohorts. Sixth, selection bias is an inherent limitation of voluntary surveys. Although the substudies were demographically similar, their recruitment methods differed: Substudy 1's on-site approach may have captured a broader attitudinal spectrum, whereas Substudy 2's remote design may be susceptible to self-selection bias, potentially overrepresenting strong viewpoints. However, the high MS PATHS enrollment rate (nearly 90% of eligible patients at our center) means our study samples were drawn from a pool encompassing nearly all patients, likely capturing diverse experiences and tempering this bias's impact. Finally, our enhanced MSPT protocol, which provides on-screen feedback and optional staff support, may have increased patient engagement disproportionately, thereby positively influencing experience ratings.

Our study findings not only advocate for the reintroduction of MSPT-like monitoring but also create a clear mandate for the targeted technological innovation of more inclusive digital tools. Future iterations should therefore prioritize accessibility by employing adaptive protocols that accommodate patients with higher disability levels or other barriers to a positive digital user experience. This includes the use of alternative motor tasks and passive smartphone- or wearable-based data collection to quantify disease activity and physical function across the entire disability spectrum, including non-ambulatory users, as exemplified by next-generation platforms (42). Ultimately, a hybrid model may emerge, synergizing periodic, high-fidelity in-clinic assessments - valued by patients in our study - with continuous, real-world passive data from wearables (54). This integrated approach could provide a more holistic picture of disease progression while aligning with patient preferences. Furthermore, the digital biomarkers captured may guide personalized remote therapeutic interventions (55), ultimately translating neuroperformance assessment into direct patient benefit.

## 5 Conclusion

This study provides real-world quantitative and qualitative insights into the prolonged use of the MSPT by both patients and physicians, extending beyond the initial implementation phase. The findings demonstrate sustained usability, acceptability, and satisfaction with the MSPT and highlight its perceived value as a clinical monitoring tool. These results can inform the future design and implementation of digital neuroperformance assessments for use in both in-clinic and remote care settings.

## Data Availability

Anonymized data will be shared with qualified investigators upon request to the corresponding author for purposes of replicating procedures and results.
