# Supplementary material for: From implementation to discontinuation: multi-year experience with the multiple sclerosis performance test as a digital monitoring tool
Source: Front Digit Health. 2025 Oct 3;7:1672732. doi: 10.3389/fdgth.2025.1672732 (PMC12531176; doi:10.3389/fdgth.2025.1672732)
Supplement: Supplementary file 1 [file Datasheet1.pdf]

SUPPLEMENTARY INFORMATION

CONTENTS

Table S1	Patient Questionnaire Items (Substudy 1) – Original Wording and Condensed Versions
Table S2	Physician Questionnaire Items (Substudy 1) – Original Wording and Condensed Versions with Descriptive Statistics
Table S3	Patient Questionnaire Items (Substudy 2) – Original Wording and Condensed Versions
Table S4	Patient Characteristics in Substudy 1 and 2
Table S5	Descriptive Results of MSPT Functional Tests Modules and NeuroQol Domains
Table S6	Association of Patient Characteristics with MSPT Experience Domains (Substudy 1): Descriptive Outcomes and Linear Regression Results
Table S7	Association of Patient Characteristics with key MSPT Discontinuation-related outcomes: Descriptive Outcomes and Logistic Regression Results
Figure S1	Association between Neuro-QoL Domain Scores and Patients' Experience and Perceptions of the MSPT

Table S1: Patient Questionnaire Items (Substudy 1) – Original Wording and Condensed Versions

#	Questionnaire item<br>(short form, re-scaled)	Original question<br>(long-version)
q1	Ease of performing the MSPT (as a whole) (0-10)	How easy or difficult is it to perform the MSPT (as a whole)? (0=difficult, 10=easy)
q2	Ease of completing the Neuro-QoL (0-10)	How easy or difficult is it to complete the Neuro-QoL questionnaire? (considering factors such as readability and question clarity) (0=difficult, 10=easy)
q3	Ease of performing the NPTs (0-10)	How easy or difficult is it to perform the NPTs (including instructions and execution)? (0 = difficult, 10 = easy)
q4	Personal use of MSPT results for self-monitoring (0-10)	To what extent do you use the results for self-monitoring? (0 = not at all, 10 = always)
q5	Usefulness of MSPT results for clinical MS monitoring (0-10)	How useful is it to incorporate the MSPT results into MS (progress) monitoring? (0=not useful; 10=useful)
q6	Conviction of MSPT results use in MS monitoring (0-10)	How convinced are you that the MSPT results are used in MS (progress) monitoring? (0 = not at all convinced, 10 = very convinced)
q7	Appropriateness of MSPT time requirement (0-10)	How do you rate the time required to complete the MSPT (as a whole)? (0 = too time-consuming, 10 = appropriate)
q8	Low physical/cognitive strain during MSPT (0-10)	Is it physically or cognitively straining / exhausting to perform the MSPT? (0 = exhausting, 10 = not exhausting)
q9	Low physical/cognitive strain during Neuro-QoL (0-10)	Is it physically or cognitively straining / exhausting to complete the Neuro-QoL questionnaire? (0 = exhausting, 10 = not exhausting)
q10	Low physical/cognitive strain during NPTs (0-10)	Is it physically or cognitively straining / exhausting to perform the functional tests (NPTs)? (0 = exhausting, 10 = not exhausting)
q11	Suitability of MSPT for independent conduct (0-10)	How well can the MSPT be performed independently? (without support) (0 = poorly, 10 = very well)
q12	Comfort of MSPT implementation (0-10)	How do you rate the comfort level in performing the MSPT? (0 = very uncomfortable, 10 = very comfortable)
q13	Good support and explanations by study staff (0-10)	How do you rate the support and explanations provided by the study staff? (0 = poor, 10 = very good)
q14	Appropriateness of quarterly MSPT conduct (0,5,10)	Do you consider quarterly MSPT assessments sufficient (if no relapse occurs)? (0=less frequent, 5=sufficient, 10=more frequent)

Abbreviations: MSPT = Multiple Sclerosis Performance Test, Neuro-QoL = Quality of Life in Neurological Disorders, NPT = Neuroperformance Test

Table S2: Physician Questionnaire Items (Substudy 1) – Original Wording and Condensed Versions with Descriptive Statistics

#	Questionnaire item (short form, re-scaled; 0= disagreement, 10=agreement)	Original question (long-version)	Central tendency & dispersion		Percentage distribution				
			Median (5-95th PCT); IQR	Mean (SD)	0-6 (%)	7-8 (%)	9-10 (%)	NPS[1]	n
q1	Ease of performing the MSPT (as a whole) (0-10)	How easy or difficult is it for patients to perform the MSPT (as a whole)? (0=difficult, 10=easy)	8.0 (5-10); 7-9	8.00 (1.63)	20%	40%	40%	20%	10
q2	Ease of completing the Neuro-QoL (0-10)	How easy or difficult is it for patients to complete the Neuro-QoL? (considering factors such as readability & question clarity) (0=difficult, 10=easy)	8.0 (7-10); 8-9	8.20 (0.92)	0%	70%	30%	30%	10
q3	Ease of performing the NPTs (0-10)	How easy or difficult is it for patients to perform the NPTs (including instructions and execution)? (0=difficult, 10=easy)	7.5 (4-10); 6-9	7.40 (1.78)	30%	40%	30%	0%	10
q4	Appropriateness of MSPT time requirement (0-10)	How do you rate the time required to complete the MSPT (as a whole)? (0=too time-consuming, 10=appropriate)	7.5 (5-10); 7-10	7.90 (1.79)	20%	40%	40%	20%	10
q5	Appropriateness of Neuro-QoL time requirement (0-10)	How do you rate the time required to complete the Neuro-QoL questionnaire? (0=too time-consuming, 10=appropriate)	8.0 (3-10); 8-9	7.70 (1.95)	20%	50%	30%	10%	10
q6	Appropriateness of NPT time requirement (0-10)	How do you rate the time required to complete the NPTs? (0=too time-consuming, 10=appropriate)	9.0 (4-10); 6-10	8.00 (2.11)	30%	10%	60%	30%	10
q7	Suitability of MSPT for independent conduct (0-10)	How well can the MSPT be performed independently (without support)? (0=poorly; 10=very well)	9.0 (5-10); 5-10	8.00 (2.31)	30%	20%	50%	20%	10
q8	Patient satisfaction with the MSPT (0-10)	Based on your experience, how satisfied are your patients with the MSPT? (0=very dissatisfied; 10=very satisfied)	7.5 (4-10); 5-9	7.20 (2.35)	40%	20%	40%	0%	10
q9	Use of MSPT results in patient monitoring (0-10)	To what extent do you use the results of the MSPT in the monitoring resp. treatment of your patients? (0=not at all; 10=always)	8.5 (3-10); 8-10	8.10 (2.18)	20%	30%	50%	30%	10
q10	Usefulness of results for MS monitoring (0-10)	How useful is it to incorporate the MSPT results into MS (progress) monitoring? (0=not useful; 10=useful)	10.0 (6-10); 9-10	9.20 (1.48)	10%	10%	80%	70%	10
q11	High sensitivity of Neuro-QoL results (0-10)	How sensitive do you consider the Neuro-Qol questionnaire results? (0=not sensitive; 10=very sensitive)	8.0 (6-10); 7-9	8.10 (1.20)	10%	50%	40%	30%	10
q12	High sensitivity of NPT results (0-10)	How sensitive do you consider the functional test (NPTs) results? (0=not sensitive; 10=very sensitive)	8.0 (7-10); 7-10	8.40 (1.26)	0%	60%	40%	40%	10
q13	Usefulness of an online (remote) MSPT (0-10)	Would an online version of the MSPT for patients to complete at home be useful? (0=not useful; 10=useful)	6.00 (0-10) 3-9;	5.80 (3.33)	60%	10%	30%	-30%	10
q14	Appropriateness of quarterly MSPT conduct (0,5,10)	Do you consider quarterly MSPT assessments sufficient (every three month)? (0=Less frequent, 5=sufficient, 10=more frequent)	5.00 (5-5); 5-10	6.00 (2.11)	0[2]: 0%	5[2]: 80%	10[2]: 20%	n.a.	10
q15	Completeness of the current MSPT Tests battery (0-10)	Are any questionnaires or tests missing? Do you believe (more) comprehensive examinations and questionnaires (e.g., medication lists, comorbidities, treatment satisfaction, etc.) are necessary for high-quality monitoring (0=yes, things are missing, 10=no, nothing missing)	9.00 (0-10); 0-10	6.00 (4.81)	40%	10%	50%	10%	10

Abbreviations: MSPT = Multiple Sclerosis Performance Test, Neuro-QoL = Quality of Life in Neurological Disorders, NPT = Neuroperformance Test

Table S3: Patient Questionnaire Items (Substudy 2) – Original Wording and Condensed Versions

#	Questionnaire item<br>(short form, re-scaled)	Original question<br>(long-version)	Condition (Addressee of each question)
q1	Negative impact of MSPT discontinuation (Yes, No)	Have you experienced any negative consequences as a result of the discontinuation of the MSPT? (Yes, No)	All patients
q2a	Reasons for negative impact of MSPT discontinuation on self-monitoring<br>▪ Absence of key feedback for validating self-monitoring of the disease<br>▪ Lack of contact with study staff<br>▪ Need to rely on other options (apps, diaries)<br>▪ Others	To what extent has the discontinuation of the MSPT negatively impacted your self-monitoring? (multiple answers are possible)<br>▪ The results of the MSPT were important in validating my own perception of my disease status.<br>▪ I miss contact with the study staff<br>▪ As a result, I now rely on other options, such as apps or diaries<br>▪ Further changes [Open text field]	If q1=yes
q2b	Reasons for no impact (or positive impact) of MSPT discontinuation<br>▪ Never used MSPT data for self-assessment<br>▪ Doctor's feedback is sufficient /more important<br>▪ Shorter visits are more valuable<br>▪ MSPT was too exhausting<br>▪ Perceived pressure to succeed and stress<br>▪ Others	Please indicate why the discontinuation of the MSPT has not impacted your self-monitoring or why it has even had a positive effect (multiple answers are possible)<br>▪ I had never used the data for my own self-assessment.<br>▪ The feedback from the doctor is sufficient or more important to me.<br>▪ Shorter visits are more valuable to me.<br>▪ The MSPT was too exhausting for me.<br>▪ I felt a “pressure to succeed” that was stressful.<br>▪ Further reasons: [Open text field]	If q1 = no
q3	Interest in resumption of MSPT-like monitoring (Yes, No)	Would you like to resume continue regular monitoring similar to the MSPT, with access to your results?<br>(Yes, No)	All patients
q4a	Suggestions/requests for resumption of MSPT-like self-monitoring<br>▪ It should stay the same<br>▪ More gamified testing desired<br>▪ Shorter execution time preferred<br>▪ More variety in the tests can be benefical<br>▪ Adaptions to limitations (e.g., alternative or language-based tests/questionnaires)<br>▪ Others	What suggestions or requests do you have for monitoring similar to the MSPT?<br>(multiple answers are possible)<br>▪ The MSPT should be conducted as it was previously.<br>▪ I would like to see a more gamified testing experience (gamification)<br>▪ I would prefer shorter visits. (Please specify the acceptable duration: [Open text field])<br>▪ Greater variety in the tests would be beneficial.<br>▪ Adaptations for specific needs or limitations would be important (e.g. alternative or language-based tests).<br>▪ Others [Open text field]	If q3 = yes
q4b	Reasons for lack of interest in resumption of MSPT-like monitoring<br>▪ Time commitment is too demanding<br>▪ Tests & questionnaires are too stressful.<br>▪ No regular MS confrontation desired<br>▪ Others	Please briefly explain your reasons for not wanting to resume a monitoring similar to the MSPT.<br>(multiple answers are possible)<br>▪ The time commitment is too demanding for me.<br>▪ The tests and questionnaires are too stressful.<br>▪ I prefer not to confront myself with my MS on a regular basis.	IF q3 = no

		▪ Others [Open text field]	
q4c	Considering of self-monitoring when certain conditions are met	Would you consider self-monitoring if certain conditions were met?<br>(Yes, No)	IF q3 = no
q4d	Conditions for self-monitoring participation	Under what conditions would you be willing to participate in self-monitoring?<br>Please specify all relevant conditions:<br>[Open text field]	IF q4c = yes
q5	Role of support to perform self-monitoring during routine visits<br>▪ Proper explanations make self-monitoring feasible for all patients.<br>▪ Support level similar to the MSPT-period is ideal<br>▪ Support upon request would suffice<br>▪ Others	Do you think all patients can easily perform self-monitoring during routine visits independently (without support)?<br>(multiple answers are possible)<br>▪ With proper explanations, self-monitoring should be possible for patients with different needs or restrictions.<br>▪ It would be ideal to have the same level of support as during the MSPT period.<br>▪ It would be sufficient to have support available on request.<br>▪ Others [Open text field]	All patients
q6	Willingness to conduct regular digital self-monitoring at home (remote)<br>(Yes, No)	Would you consider performing regular digital monitoring independently from home (remote)?<br>(Yes, No)	All patients
q7a	Acceptable frequency of a remote digital self-monitoring<br>▪ Weekly<br>▪ Every two weeks<br>▪ Monthly<br>▪ Less than monthly<br>▪ Others	How often would a remote digital monitoring be acceptable to you?<br>▪ Once per week<br>▪ Every two weeks<br>▪ Once per month<br>▪ Less frequent than once per month<br>▪ Further suggestions: [Open text field]	IF q6 = yes
q7b	Barriers to home-based digital self-monitoring<br>▪ Time commitment is too high<br>▪ Difficult to integrate into daily routine<br>▪ Tests & questionnaires are too stressful.<br>▪ No regular MS confrontation desired<br>▪ Concern about data security and privacy<br>▪ Own control over access to data and test results wanted<br>▪ Internet connectivity issues.<br>▪ Others	What prevents you from conducting digital self-monitoring at home?<br>▪ The time commitment is too demanding for me.<br>▪ Difficulty to integrate monitoring into my daily routine.<br>▪ The tests and questionnaires are too stressful.<br>▪ I prefer not to confront myself with my MS on a regular basis.<br>▪ I am concerned about data security and privacy.<br>▪ I want to control who has access to my data and test results.<br>▪ Internet connectivity issues.<br>▪ Further reasons: [Open text field]	IF q6 = no

Table S4: Patient Characteristics in Substudy 1 and 2.

		Substudy 1 (2019-2020)	Substudy 2 (2024)	p-value (Chi-Square Tests)
		n=200	n=144	
Age (years)	Median (IQR)	43.0 (35.0-53.0)	n.a.	p < 0.001
	≤40 years, n (%)	86 (43.0%)	36 (25.0%)	
	41-60 years, n (%)	97 (48.5%)	78 (54.2%)	
	>60 years, n (%)	17 (8.5%)	30 (20.8%)	
Sex	Female, n (%)	159 (79.5%)	115 (79.9%)	p=0.9346
	Male, n (%)	41 (20.5%)	29 (20.1%)	
Experience with the MSPT (years)	Median (IQR)	1.5 (1.00-1.75)	n.a.	-
	0-2 years, n (%)	200 (100.0%)	47 (32.6%)	
	3-4 years, n (%)	0 (0%)	54 (37.5%)	
	5-6 years, n (%)	0 (0%)	43 (29.9%)	
Disease Duration (years)	Median (IQR)	9.0 (5.0-14.0)	n.a.	p= 0.1080
	≤10, n (%)	119 (59.5%)	52 (48.6%)	
	10-15, n (%)	37 (18.5%)	30 (28.0%)	
	>15, n (%)	44 (22.0%)	25 (23.4%)	
Disability - EDSS	Median (IQR)	2 (1.50-3.75)		
	EDSS 0-1.5, n (%)	56 (28.0%)		
	EDSS 2-3.5, n (%)	94 (47.0%)		
	EDSS 4-5.5, n (%)	36 (18.0%)		
	EDSS 6+, n (%)	14 (7.0%)		
Disability - Number of patient-reported Symptoms[1]	Median (IQR)		4.0 (2.0-6.0)	
	0-2 Symptoms		37 (25.7%)	
	3-5 Symptoms		62 (43.1%)	
	6-8 Symptoms		30 (20.8%)	
	9+ Symptoms		15 (10.5%)	
MS Type	RRMS	187 (93.5%)		
	CIS	2 (1.0%)		
	PPMS	3 (1.5%)		
	SPMS	8 (4.0%)		
Current DMT	No DMT	20 (10%)		
	B-cell Depletion Therapy	32 (16.0%)		
	Induction Therapy	17 (8.5%)		
	VCAM-1 Blocking Therapy	12 (6.0%)		
	S1PR Modulating Therapy	40 (20.0%)		
	Platform Oral Therapy	46 (23.0%)		
	Platform Therapy	32 (16.0%)		
	Other	1 (0.5%)		
PDDS	Median (IQR)	1.0 (0.0-2.0)		
Education (years)	Median (IQR)	12 (10-16)		
	≤9 years (Basic Education)	19 (9.5%)		
	10-12 years (Intermediate)	87 (43.5%)		
	≥13 years (Higher)	94 (47.0%)		
Previous MSPTs (number)	Median (IQR)	5.0 [3.0-6.0]		
	1-3, n (%)	55 (27.5%)		
	4-6, n (%)	105 (52.5%)		
	7+, n (%)	40 (20.0%)		

Socio-demographic and disease-specific variables for Substudies 1 and 2 are shown. Age, disease duration, sex, MSPT experience, and a disability proxy were captured in both surveys. Because Substudy 2 was conducted anonymously online, fewer variables were collected, values are reported only categorically, and linkage to Substudy 1 records is impossible; participant overlap therefore remains uncertain. MSPT module-specific outcomes for Substudy 1 are provided in Table S5

[1] Substudy 2 variables were categorized as follows: age in 10-year strata, MSPT experience in 2-year strata, and disease duration in 5-year strata - the most granular classification available for analysis (Figure 4).

[2] The total number of symptoms was derived from yes/no responses to the following list: bladder dysfunction, bowel dysfunction, cognitive impairment (e.g. attention, concentration, memory), depression, dizziness/balance disturbance, fatigue, gait disturbance, pain (including headache), sensory disturbance, spasticity, speech/language disturbance, visual disturbance, and “other” symptoms.

Abbreviations: CIS = Clinically Isolated Syndrome, DMT = Disease-Modifying Therapy, IQR = Interquartile Range, MSPT = Multiple Sclerosis Performance Test, PPMS = Primary Progressive Multiple Sclerosis, RRMS = Relapsing-Remitting Multiple Sclerosis, SPMS = Secondary Progressive Multiple Sclerosis

Table S5: Descriptive Results of MSPT Functional Tests Modules and NeuroQol Domains

	MSPT results n=200
Neuroperformance Test Modules	
CST completed, n (%)	196 (98.0%)
CST result[2] (number), median [IQR]	41.0 (33.0-46.0)
CST result[2] (z-score) median [IQR]	0.15 (-0.55-0.72)
PST completed, n (%)	200 (100%)
PST result[2] (number), median [IQR]	54.5 (45.0-64.0)
PST result[2] (z-score), median [IQR]	0.24 (-0.51-0.95)
WST completed, n (%)	196 (98.0%)
WST result[1] (seconds), median [IQR]	4.37 (3.96-5.22)
WST result[2] (z-score), median [IQR]	1.27 (0.62-1.73)
MDT completed, n (%)	200 (100%)
MDT result[1] - Average Both Hands (seconds), median [IQR]	21.71 (19.05-25.22)
MDT result[2] - Average Both Hands (z-score), median [IQR]	0.81 (0.05-1.39)
Completed functional tests (number)	
4 (CST, PST, WST and MDT), n (%)	192 (96.0%)
3, n (%)	8 (4.0%)
0-2, n (%)	0 (0%)
Completion Time (seconds), median [IQR]	878 (804-1018)
NeuroQol Module	
Mental Domains:	
Depression[1] (T-Score), median [IQR]	44.02 (37.86-49.43)
Anxiety[1] (T-Score), median [IQR]	48.94 (38.56-54.94)
Stigma[1] (T-Score), median [IQR]	43.57 (36.56-50.07)
Cognitive Function[2] (T-Score), median [IQR]	51.09 (46.08-56.76)
Physical Domains:	
Upper Extremity Function[2] (T-Score), median [IQR]	56.60 (39.02-56.60)
Lower Extremity Function[2] (T-Score), median [IQR]	50.88 (44.40-62.06)
Sleep Disturbance[1] (T-Score), median [IQR]	47.50 (40.22-54.86)
Fatigue[1] (T-Score), median [IQR]	43.16 (39.32-50.45)
Social Domains:	
Ability to Participate in Social Roles & Activities[2] (T-Score), median [IQR]	48.29 (43.78-54.31)
Satisfaction with Social Roles & Activities[2] (T-Score), median [IQR]	48.27 (44.24-54.15)
Composite Summary Scores[3]:	
Mental Summary T-Score[1], median [IQR]	45.96 (39.44-51.81)
Physical Summary T-Score[1], median [IQR]	46.55 (40.99-54.06)
Social Summary T-Score[1], median [IQR]	50.79 (44.36-55.86)
Overall T-Score[1], median [IQR]	46.38 (41.53-52.69)
Completed Neuro-QoLs	
NeuroQol performed (pwMS), n (%)	99 (49.5%)
Completion Time (seconds), median [IQR]	338 (279-435)

Descriptive results and completion rates of the MSPT modules, including Z-scores and raw scores for the CST, PST, WST, and MDT, as well as T-scores for Neuro-QoL domains.

[1] Higher scores indicate 'worse' self-reported health (Neuro-QoL) or neuro-performance (functional tests)
[2] Higher scores indicate 'better' self-reported health (Neuro-QoL) or neuro-performance (functional tests)
[3] Domain-specific summary scores were calculated as composite measures by averaging the associated Neuro-QoL T-scores as (unweighted) arithmetic means. For domains where lower T-scores indicate worse health, scores were reverse-scored (Reversed T-score = 100 - Original T-score) to ensure consistent directionality (higher scores = worse health). While providing a practical summary of the data and serving as practical proxies for broader constructs, they have not been formally validated and should be interpreted with caution.

Abbreviations: CST = Cognitive Processing Speed Test, PST = Pegboard Speed Test, WST = Walking Speed Test, MDT = Manual Dexterity Test, IQR = Interquartile Range, MSPT = Multiple Sclerosis Performance Test, Neuro-QoL = Quality of Life in Neurological Disorders, pwMS = People with Multiple Sclerosis

Table S6: Association of Patient Characteristics with MSPT Experience Domains (Substudy 1): Descriptive Outcomes and Linear Regression Results

		Task Strain (PC1)			Task Ease & Independence (PC2)			Outcome Utility & Timing (PC3)			Support & Comfort (PC4)		
		NPS Description	Linear Regression		NPS Description	Linear Regression		NPS Description	Linear Regression		NPS Description	Linear Regression	
		Wtd. Mean (q8-q10)	b	p-value	Wtd. Mean (q1-q3, q11)	b	p-value	Wtd. Mean (q4-q7,q14)	b	p-value	Wtd. Mean (q12-q13)	b	p-value
Age (years)	continuous, per year	-	-0.119	0.057	-	-0.243	0.001*	-	0.142	0.048	-	-0.101	0.162
	≤ 40 years	69.15%	Ref	-	85.69%	Ref	-	38.15%	Ref	-	80.33%	Ref	-
	41 – 60 years	53.36%	-0.057	0.446	69.20%	-0.219	0.003	50.19%	0.157	0.037*	76.29%	-0.074	0.327
	> 60 years	44.79%	-0.114	0.132	60.67%	-0.193	0.009	60.71%	0.107	0.154	76.47%	-0.100	0.185
Sex	Male	54.40%	Ref		78.43%	Ref		37.71%	Ref		80.95%	Ref	
	Female	60.76%	0.077	0.288	74.82%	-0.004	0.957	48.02%	0.019	0.794	77.28%	0.000	0.995
MSPT Experience (years)	continuous, per year	-	0.062	0.394	-	-0.157	0.028	-	0.094	0.190	-	-0.037	0.608
	0-2 years	59.43%	-	-	75.55%	-	-	45.91%	-	-	78.04%	-	-
	3-4 years	-	-	-	-	-	-	-	-	-	-	-	-
	5-6 years	-	-	-	-	-	-	-	-	-	-	-	-
Disease Duration (years)	continuous, per year	-	0.032	0.654	-	-0.326	0.000	-	0.015	0.836	-	-0.050	0.486
	≤ 10 years	63.86%	Ref		80.21%	Ref		46.51%	Ref	-	81.67%	Ref	-
	10-15 years	58.60%	-0.024	0.753	77.64%	-0.074	0.310	41.94%	-0.037	0.617	76.06%	-0.007	0.930
	> 15 years	47.73%	-0.033	0.663	61.10%	-0.212	0.004	47.61%	0.003	0.970	69.50%	-0.128	0.087
Disability (EDSS)	continuous, per year	69.08%	-0.222	0.002	-	-0.290	0.001	-	0.091	0.209	-	-0.158	0.028
	EDSS 0-1.5	75.06%	Ref	-	83.14%	Ref	-	40.02%	Ref	-	78.57%	Ref	
	EDSS 2-3.5	18.99%	0.088	0.276	80.91%	-0.091	0.280	46.29%	0.025	0.775	81.51%	-0.012	0.891
	EDSS 4+	69.08%	-0.290	<0.001	56.96%	-0.266	0.002	51.78%	0.130	0.129	70.84%	-0.210	0.014

Standardized regression coefficients (b) and corresponding p-values from linear regression models examining associations between basic patient characteristics (independent variables) and four aggregated domains of MSPT experience (dependent variables). The independent domains represent principal components (PC) derived through principal component analysis: Strain (PC1), Task Ease & Independence (PC2), Outcome Utility & Timing (PC3), and Support & Comfort (PC4). To optimize normality and achieve standardization, PC1-PC4 underwent transformations (constant shift, reflections, logarithmic transformation) and z-score normalization before regression.

For descriptive context, the mean Net Promoter Scores (NPS) per patient subgroup are provided, calculated as weighted means using the NPS and principal component loadings from q1–q14 that are primarily relevant to the respective domain. The categorization of patient characteristics variables aligns with the reporting constraints of the anonymous Substudy 2 survey, where patient data were primarily available in categorical form. Table S7 provides a subgroup analysis of key Substudy 2 outcomes using the same categorization approach.

Abbreviations: b = Standardized regression coefficient (beta), EDSS = Expanded Disability Status Scale, NPS = Net Promoter Score, MSPT = Multiple Sclerosis Performance Test, Ref. = Reference category, q1–q14 = Questionnaire items 1 to 14 from the patient experience questionnaire in Substudy 1 (Table S1), Wtd = Weighted

Table S7: Association of Patient Characteristics with key MSPT Discontinuation-related outcomes: Descriptive Outcomes and Logistic Regression Results.

Substudy 2		Patient-perceived impact of MSPT discontinuation (q1) (yes vs no)			Interest in resuming MSPT-like digital disease monitoring (q3) (yes vs no)			Willingness to perform digital self-monitoring at home (remote) (q6) (yes vs no)		
		Description	Logistic Regression		Description	Logistic Regression		Description	Logistic Regression	
		% Yes	OR	95%-CI	% yes	OR	95%-CI	% yes	OR	95%-CI
Age (years)	continuous, per year	-	-	-	-	-	-	-	-	-
	≤ 40 years	66.7%	Ref	-	88.9%	Ref		86.1%	Ref	
	41 – 60 years	61.5%	0.800	0.349-1.834	85.9%	0.761	0.225-2.577	79.5%	0.625	0.210-1.864
	> 60 years	56.7%	0.654	0.240-1.779	80.0%	0.500	0.127-1.970	66.7%	0.323	0.096-1.084
Sex	Male	58.6%			79.3%	Ref		82.8%	Ref	
	Female	62.6%	1.182	0.515-2.710	87.0%	1.739	0.609-4.968	77.4%	0.713	0.248-2.054
MSPT Experience (years)	continuous, per year	-	-	-	-	-	-	-	-	-
	<2 years	53.2%	Ref	-	??	Ref	-	83.0%	Ref	-
	3-4 years	63.0%	1.496	0.675-3.316	79.6%	0.802	0.292-2.198	77.8%	0.718	0.265-1.942
	5-6 years	69.8%	2.031	0.853-2.015	95.3%	4.205	0.840-21.044	74.4%	0.597	0.214-1.661
Disease Duration (years)	continuous, per year	-	-	-	-	-	-	-	-	-
	≤ 10 years	63.5%	Ref	-	88.5%	Ref	-	80.8%	Ref	Ref
	10-15 years	73.3%	1.583	0.590-4.247	90.0%	1.174	0.271-5.081	86.7%	1.548	0.440-5.448
	> 15 years	52.0%	0.624	0.237-1.640	88.0%	0.957	0.219-4.186	80.0%	0.952	0.297-3.157
Disability (Number of patient-reported Symptoms)	continuous, per year	-	-	-	-	-	-	-	-	-
	0-2	56.8%	Ref		89.2%	Ref		89.2%*	Ref	
	3-5	59.7%	1.128	0.494-2.573	80.6%	0.505	0.150-1.700	77.4%*	0.416	0.126-1.375
	>5	68.9%	1.687	0.682-4.176	88.9%	0.970	0.241-3.906	71.1%*	0.298	0.088-1.012

Odds ratios (OR) with 95% CIs from logistic regression models assessing associations between core patient characteristics (independent variables) and key MSPT discontinuation related outcomes (dependent variables) in Substudy 2. All 95% CIs for ORs in the table correspond to $p > 0.05$. Asterisks (*) indicate statistically significant Cochran–Armitage test for trend ($p < 0.05$). For descriptive context, the percentage of positive responses (% yes) per subgroup is provided. Analyses are based on data from Substudy 2, where patient characteristics were primarily categorical due to the anonymous online survey design.

Abbreviations: OR = Odds Ratio; CI = Confidence Interval; EDSS = Expanded Disability Status Scale; MSPT = Multiple Sclerosis Performance Test; Ref. = Reference Category; Q1–Q6 = Patient questionnaire items 1–7 from Substudy 2 (Table S2).

Figure S1: Association between Neuro-QoL Domain Scores and Patients' Experience and Perceptions of the MSPT

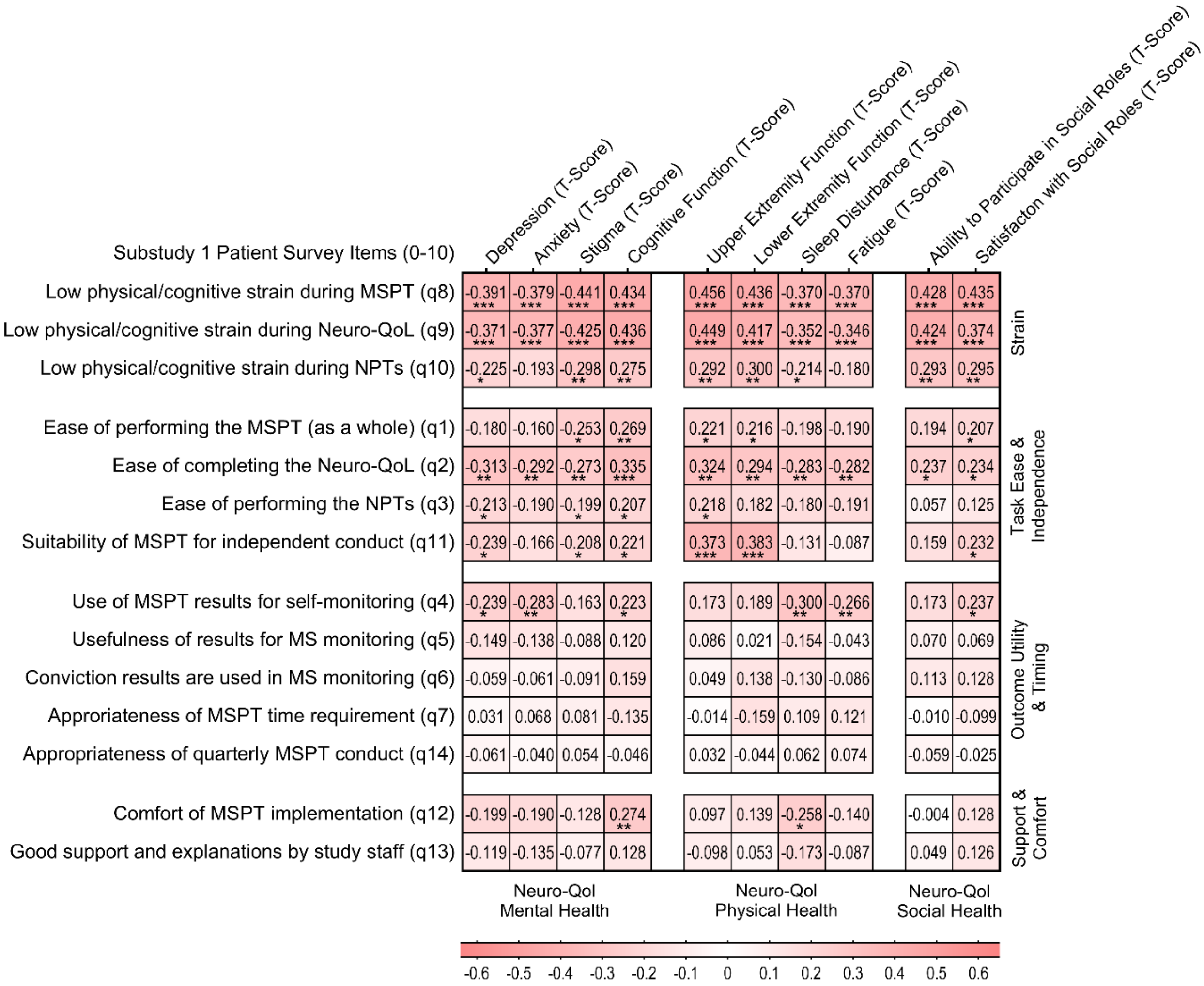

Heatmap illustrating correlation patterns (Spearman's rho) between MSPT patient experience items and Neuro-QoL domains. A detailed breakdown of 10 individual Neuro-QoL domains is provided, while Figure 3 presents only results for aggregated Neuro-Qol summary score proxies. Neuro-QoL T-scores are standardized to a reference population (mean = 50, SD = 10). The interpretation of Neuro-QoL scores is dependent upon the specific domain being assessed. Higher T-scores signal poorer quality of life/greater impairment for Depression, Anxiety, Stigma, Sleep Disturbance, and Fatigue, whereas higher scores indicate better quality of life/functioning for Cognitive Function, Upper- and Lower-Extremity Function, Ability to Participate in Social Roles, and Satisfaction with Social Roles.Statistically significant associations are marked with asterisks: * $p < 0.05$, ** $p < 0.01$, *** $p < 0.001$
